# Throat and nasal swabs for molecular detection of respiratory viruses in acute pharyngitis

**DOI:** 10.1186/s12985-015-0408-z

**Published:** 2015-10-29

**Authors:** Mohsin Ali, Sangsu Han, Chris J. Gunst, Steve Lim, Kathy Luinstra, Marek Smieja

**Affiliations:** Icahn School of Medicine at Mount Sinai, New York, NY USA; Faculty of Medicine, University of Ottawa, Ottawa, ON Canada; Department of Immunology, University of Toronto, Toronto, ON Canada; Faculty of Dentistry, University of Toronto, Toronto, ON Canada; St. Joseph’s Healthcare, St. Luke’s Wing, L-424, 50 Charlton Avenue East, Hamilton, ON Canada L8N 4A6; Department of Pathology and Molecular Medicine, McMaster University, Hamilton, ON Canada

**Keywords:** Pharyngitis, Throat swab, Nasal swab, Respiratory viruses, Detection

## Abstract

**Background:**

Detection of specific respiratory viruses is important for surveillance programs, where nasopharyngeal or nasal swabs have traditionally been used. Our objective was to determine whether sampling with a throat swab provides *incremental* benefit—when used in conjunction with a nasal swab—to detect respiratory viruses among patients with acute pharyngitis in the outpatient setting.

**Findings:**

Among 83 university students with acute pharyngitis, we detected respiratory viruses with molecular assays on two samples collected per student: with a flocked nasal mid-turbinate swab and a rayon throat swab. Forty-eight (58 %) patients had virus-positive samples, with 49 virus positives detected by either swab (one patient had a dual viral co-infection). The most common viruses were rhinovirus, coronavirus, and influenza A virus. Specifically, 29 virus positives were detected by both swabs, 14 exclusively by the nasal swab, and six exclusively by the throat swab. The additional six virus positives detected by the throat swab corresponded to an absolute increase in viral detection of 7.1 % (95 % CI: 1.2–12.9 %); the specific viruses detected were four rhinoviruses and two coronaviruses.

**Conclusions:**

The flocked nasal swab samples respiratory viruses well, even among patients whose primary complaint is a sore throat. The rayon throat swab has modest incremental value over and above using the flocked nasal mid-turbinate swab alone, which suggests that while throat swabs alone would not be adequate for respiratory viral surveillance, they may have value as a supplementary test.

Acute pharyngitis is one of the most common illnesses for which patients visit primary-care physicians, accounting for 1–2 % of all visits [1, 2]. For these patients, throat swabs are primarily used to detect *Streptococcus pyogenes*, otherwise known as Group A beta-hemolytic streptococcus (GABHS); however, most cases of acute pharyngitis are due to viruses. Detection of specific viruses, especially influenza A, is important for respiratory viral surveillance programs, where nasopharyngeal or nasal swabs have traditionally been used. Some recent research, however, suggests that incorporating throat swabs into these programs may increase sensitivity to detect respiratory viruses [3, 4]. However, to our knowledge, no studies have quantified the incremental benefit that throat swabs could provide for surveillance of respiratory viruses.

Our primary objective was to determine whether sampling with a throat swab provides *incremental* benefit—when used in conjunction with a nasal swab—to detect respiratory viruses among adults with acute pharyngitis. We used a flocked mid-turbinate nasal swab, instead of a flocked nasopharyngeal swab, for several reasons. First, our study was done in an outpatient setting, where nasopharyngeal sampling for respiratory viruses does not routinely occur. In addition, previous research among adults has shown that flocked nasal mid-turbinate swabs are as sensitive for respiratory virus testing as flocked nasopharyngeal swabs, and since nasal mid-turbinate swabs are more comfortable and acceptable for patients, these swabs are preferred for sample collection in outpatient studies [5]. There is also comparable sampling capacity between flocked nasopharyngeal and flocked mid-turbinate nasal swabs in terms of cell counts and levels of beta-actin, a measure of sampling adequacy [6]. Finally, nasal swabs are routinely used for the purpose of respiratory viral surveillance (the focus of our study), such as in the community setting for influenza. In terms of sample size, we specified *a priori* an absolute incremental benefit of 10 % for viral detection (i.e., 10 extra virus-positive samples detected by only the throat swab per 100 patients) to be significant for the purpose of respiratory viral surveillance. To show this level of effect, we required 77 patients, assuming a two-sided test, α = 0.05 and power of 80 %. Our secondary objective was to describe the viruses detected among patients with acute pharyngitis using molecular testing, which is not routinely done clinically. The study protocol was approved by the Research Ethics Board at St. Joseph’s Healthcare, Hamilton, Ontario, Canada.

From September 2011 through March 2013, we recruited students presenting to McMaster University’s campus health clinic who were at least 17 years old, reported a sore throat, presented within three days of symptom onset (to maximize viral detection) [7], and did not have concurrent serious illness, as judged by their health-care provider. After providing consent, patients completed a questionnaire regarding onset of illness and severity of signs and symptoms, adapted from a previously validated survey [8]. Trained study staff then sampled the patient’s left side of their throat and tonsils with a rayon throat swab (Copan Italia); the left nostril was subsequently sampled using a flocked mid-turbinate nasal swab (FLOQSwabs; Copan Italia). Both swabs were placed into universal transport medium (UTM; Copan Italia) and frozen at −80 °C until analysis. Seven days later, patients were e-mailed a follow-up survey regarding the impact of their illness on their day-to-day lives.

Specimens were batch extracted using the NucliSENS easyMAG assay (bioMérieux Canada; St. Laurent, Québec) and tested for respiratory viruses using the xTAG respiratory virus panel (RVP) version 1 (RVPv1, Luminex; Austin, TX), which detects 16 virus types and subtypes [9]; two laboratory-developed multiplex real-time polymerase chain reaction (PCR) assays for adenovirus [10], metapneumovirus [11], respiratory syncytial virus [12], and influenza A and B and parainfluenza 1–3 [13] used by the Hamilton Regional Laboratory Medicine Program for routine respiratory virus diagnosis; and a reverse-transcriptase PCR for enterovirus and rhinovirus [14]. In addition, information regarding detection of beta-hemolytic streptococcal species (group A, C and G)—as diagnosed by the treating clinician during routine care—was also gathered per patient from the clinic, which did not use any of the specimens gathered from our study. As per standard of care, the clinic used rapid antigen detection tests for GABHS (Rapid Response Strep-A, BTNX; Markham, Ontario) and/or anaerobic culture on 5 % sheep-blood agar plates followed by Lancefield grouping (PathoDx Strep Grouping, Oxoid; Nepean, Ontario). Furthermore, *S. pyogenes* was detected using the throat swabs collected in our study with PCR, using primers that have shown high sensitivity and specificity [15].

Eighty-three patients participated, of whom 60 (72 %) had a respiratory virus and/or beta-hemolytic streptococci detected in at least one sample. As shown in Table 1, 48 patients had virus-positive samples and 20 had beta-hemolytic streptococci-positive samples (17 GABHS and three group C); there were eight viral–streptococcal co-infections and one dual viral co-infection. The most common viruses detected were rhinovirus/enterovirus (22 patients, of whom 18 were confirmed as rhinovirus, and the remainder could not be resolved and were assumed to be rhinovirus), coronavirus (10 patients), and influenza A (8 patients). There were no salient differences regarding clinical presentation and one-week follow-up between patients detected with beta-hemolytic streptococci and various viruses (Table 2).

**Table 1:** Infectious agents detected among 83 university students with acute pharyngitis

	Flocked nasal swab sample	Rayon throat swab sample	Either sample
Rhinovirus^a^	18	16	22
Coronavirus	8	7	10
Influenza A virus	8	8	8
Metapneumovirus	4	2	4
Respiratory syncytial virus	2	0	2
Adenovirus	1	1	1
Parainfluenza virus	1	1	1
Influenza B virus	1	0	1
No. of samples (patients) with viruses detected^b^	43 (43)	35 (34)	49 (48)
No. of patients with β-hemolytic streptococci^b,c^			20

^a^Of 22 patients with enterovirus/rhinovirus-positive samples, 18 could be unambiguously subtyped with PCR and sequencing as rhinoviruses

^b^Eight patients had a viral–streptococcal co-infection. One patient had a viral co-infection of rhinovirus and coronavirus HKU1

^c^Beta-hemolytic streptococci were detected by rapid antigen detection (group A) and/or culture (group A, C, and G) outside our study, during routine clinical care. Group A species were also detected within the study using PCR

**Table 2:** Clinical presentation and follow-up of 83 university students with acute pharyngitis

	Virus only				Streptococcal ± virus^c^	
	E/R^a^	Flu	Corona-virus^a^	Other^b^		Negatives
No. patients at presentation^a^	19	8	9	5	20	23
Symptom duration, median no. of days (IQR)	3 (1)	3 (0)	3 (1)	3 (0)	2 (2)	2 (1)
Symptoms, n (%)						
Sore throat^d^	19 (100)	8 (100)	9 (100)	5 (100)	20 (100)	23 (100)
Cough	16 (84)	8 (100)	7 (78)	4 (80)	13 (65)	15 (65)
Fever	12 (63)	8 (100)	3 (33)	5 (100)	18 (90)	9 (39)
Sneezing	14 (74)	6 (75)	7 (78)	4 (80)	7 (35)	7 (30)
Runny nose	18 (95)	8 (100)	7 (78)	4 (80)	13 (65)	12 (52)
Headache	13 (68)	7 (88)	5 (56)	5 (100)	14 (70)	13 (57)
Sinus pain/pressure	12 (63)	7 (88)	5 (56)	3 (60)	10 (50)	8 (35)
Cervical lymphadenopathy	10 (53)	6 (75)	3 (33)	3 (60)	15 (75)	13 (57)
No. (%) prescribed antibiotic	3 (16)	0 (0)	1 (11)	1 (20)	15 (75)	8 (35)
No. patients at one-week follow-up	19	7	9	6	19	21
Symptom duration, median no. of days (IQR)	4 (4)	4 (2)	7 (3)	4 (2)	3 (3)	7 (3)
No. (%) who missed ≥ 1 day of school	5 (26)	5 (71)	4 (44)	3 (50)	13 (68)	6 (29)

*Abbreviations*: *E/R* Enterovirus/rhinovirus, *IQR* Interquartile range

^a^One patient with co-infection of rhinovirus and coronavirus HKU1 was analyzed in both columns

^b^Includes patients with adenovirus (*n* = 1), metapneumovirus (*n* = 2) and respiratory syncytial virus (*n* = 2)

^c^Includes patients with group A (*n* = 17) and group C (*n* = 3) beta-hemolytic streptococci, with five and three viral co-infections, respectively. No group G beta-hemolytic streptococci were detected

^d^By definition, this proportion is 100 % given study inclusion criteria

Of 49 viruses detected with either the flocked mid-turbinate nasal or throat swabs, 29 were detected by both swabs, 14 were detected exclusively by the nasal swab, and six were detected exclusively by the throat swab, demonstrating that the flocked nasal swab sampled viruses well even among patients whose primary complaint was a sore throat. Results between the two swabs were concordant for 76 % (63/83) of patients, with a moderate level of agreement (Cohen’s kappa, 0.52; 95 % CI, 0.34–0.70).

However, since rayon throat swabs detected six virus-positive patients who were negative in nasal swab samples, there was a small *incremental* value to its use, specifically, an absolute increase in viral detection of 7.1 % (95 % CI, 1.2–12.9 %). The specific viruses detected were four rhinoviruses and two coronaviruses. This modest incremental benefit aligns with recent research on children and adults with influenza-like illness demonstrating that sampling the throat in conjunction with the nasopharynx or nose can improve sensitivity for viral detection [3, 4]. This may be if the optimal site for detection (throat, nose, or nasopharynx) varies by virus, as shown in one previous study among patients with influenza-like illness and severe acute respiratory illness, which found higher sensitivity for detection for adenovirus and influenza A in the throat [4]. Therefore, sampling with throat swabs may be valuable in the context of respiratory viral surveillance, and a cost-effective approach could be to pool nasal and throat swabs together for combined processing and amplification [13, 16].

Our study has some limitations. Without a control group of patients without acute pharyngitis, we could not distinguish infection unrelated to the presenting illness, and thus cannot infer causation. For example, it may be that the viruses detected in the nose did not cause pharyngitis. Thus, our findings pertain more to the setting of respiratory viral surveillance, where the focus is on detection rather than diagnosis. Second, the throat and nasal swabs we used were made of different materials (rayon vs. flocked nylon, respectively). Some previous research suggests that flocked throat swabs may be superior to rayon throat swabs for respiratory viral detection. A study of 223 newly admitted elderly inpatients in Norway that tested oropharyngeal and nasopharyngeal specimens for respiratory viruses using real-time PCR found 4.8-fold higher viral load (95 % CI, 1.3–17, *p* = 0.017) with flocked swabs compared to rayon swabs, regardless of sampling site [17]. This suggests that the incremental benefit we estimated for respiratory viral detection using the rayon throat swab may increase with a flocked throat swab. Thus, swab material and other relevant factors regarding swab design—including swab length and shape—require further study. Finally, as noted above, the optimal site for detection may vary by virus. We were unable to detect such differences due to our small sample size, and this also remains an area for future research.

In summary, we evaluated rayon throat and flocked mid-turbinate nasal swabs for viral detection among young adults with acute pharyngitis. Respiratory viruses were detected in the majority, particularly rhinovirus, coronavirus, and influenza A. The nasal swab sampled respiratory viruses well, even among patients whose primary complaint was a sore throat. We found modest incremental benefit for viral detection with the throat swab over and above the nasal swab alone, which suggests that while throat swabs alone would not be adequate for respiratory viral surveillance, they may have value as a supplementary test.

## Ethics, consent and permissions

Research Ethics Board approval (reference number: RP #11-3594) from St. Joseph’s Healthcare Hamilton Research Ethics Board, Hamilton, Ontario, Canada.

## Abbreviations

GABHS: Group A beta-hemolytic streptococcus
PCR: Polymerase chain reaction
RVP: Respiratory virus panel
